# Seropositivity and flight-associated risk factors for SARS-CoV-2 infection among asylum seekers arriving in Berlin, Germany – a cross-sectional study

**DOI:** 10.3389/fpubh.2023.1134546

**Published:** 2023-06-12

**Authors:** Ariadne Brandt, Lena Breucker, Jan Keller, Victor Max Corman, Norma Bethke, Joachim Seybold

**Affiliations:** ^1^Medical Directorate, Charité – Universitätsmedizin Berlin, Corporate Member of Freie Universität Berlin, Humboldt-Universität zu Berlin, Berlin, Germany; ^2^Department of Education and Psychology, Freie Universität Berlin, Berlin, Germany; ^3^Institute of Virology, Charité – Universitätsmedizin Berlin, Freie Universität Berlin, Humboldt-Universität zu Berlin, Berlin, Germany; ^4^German Centre for Infection Research, Partner Site Charité, Berlin, Germany; ^5^Labor Berlin – Charité Vivantes GmbH, Berlin, Germany

**Keywords:** SARS-CoV-2, refugees, seropositivity, hygiene behavior, prevalence, viral infection

## Abstract

**Introduction:**

Refugees and asylum seekers might be at increased risk of SARS-CoV-2 infection due to precarious living conditions during flight.

**Methods:**

Between March 24th and June 15th 2021, we conducted a cross-sectional study among adult asylum seekers arriving in Berlin. Each participant was tested for acute SARS-CoV-2 infection with a nasopharyngeal swab using reverse transcriptase PCR (rt-PCR), and for anti-SARS-CoV-2-S1 IgG antibodies using ELISA. Seropositivity, antibody avidity, and data on flight history were used to categorize individuals into two groups according to the estimated time of infection before or during flight. Sociodemographic characteristics, COVID-19 related symptoms, hygiene behaviors, and living conditions during transit were assessed using two self-report questionnaires.

**Results:**

Among 1041 participants (34·5% female, mean age 32·6 years), most frequently reported countries of origin were Moldova (20·5%), Georgia (18·9%), Syria (13·0%), Afghanistan (11·3%), and Vietnam (9·1%). Seropositivity rate was 25·1% and incidence rate of acute SARS-CoV-2 infection was 2·8%. A higher likelihood for seropositivity was observed in women (OR [95%CI]=1·64 [1·05-2·57]) but reduced by frequent hygiene behaviors (OR [95%CI]=0·75 [0·59-0·96]) or traveling by plane (OR [95%CI]=0·58 [0·35-0·96]). Other associated factors were lower educational level, accommodation in refugee shelters, traveling with children or by foot, and COVID-19 information seeking.

**Conclusion:**

Flight-associated risk factors such as accommodation in a refugee shelter and poor hygiene behaviors are associated with an elevated risk of infection, which should be addressed by public health interventions.

**Clinical trial registration:**

[https://doi.org/10.1186/ISRCTN17401860], identifier [17401860].

## Introduction

In March 2020, the United Nations Refugee Agency (UNHCR) expressed their concern that the COVID-19 pandemic could particularly affect vulnerable groups such as refugees and asylum seekers (1). These individuals face many perils during flight. Flight is defined in line with the UNHCR as the act of leaving one’s country or place of origin due to persecution, war, famine, violence, or natural disasters. Living conditions while fleeing, both during transit and upon arrival in host countries, are often characterized by overcrowded accommodation, limited access to sanitation and healthcare services, which might contribute to an increased risk of exposure (2, 3). Simultaneously, public health campaigns often neglect refugees in transit resulting in lacking knowledge on protective hygiene measures and misinformation on infectious diseases (4). Several observational and modeling studies have found an increased risk of SARS-CoV-2 infection among refugees in camps or shelters, presumably due to overcrowding of facilities, and insufficient outbreak management strategies (5–7). Further possible risk-factors for the spread of SARS-CoV-2 might include the mode of transportation during flight; available resources including face masks; and hygiene literacy regarding basic knowledge on the prevention of infectious disease. However, data on asylum seekers or refugees in transit and specific transit-related infection risks of this hard-to-reach population is limited. A systematic review reported the incidence risk of SARS-CoV-2 for migrants until June 2020 to be anywhere between 0.1% and 21.2% depending on the occurrence of outbreaks (5). SARS-CoV-2 prevalence rates within these populations may be deemed difficult to interpret as testing is not readily available in shelters or during transit and many displaced individuals are reluctant to seek out medical help (5, 8). More evidence on asylum seekers’ exposure to SARS-CoV-2 infections is warranted.

Germany belongs to one of the five countries hosting most refugees worldwide (9). In reaction to European border closings and restricted mobility at the beginning of the pandemic, many refugees were stranded in provisionary camps close to borders and the influx of asylum seekers to Germany dropped remarkably in March 2020 (10, 11). The number of registered asylum applications has regained pre-pandemic levels around June 2021. About 150,000 asylum applications were received between January and October 2021, of which 6.3% were registered in the state of Berlin (10). Most frequent countries of origin among asylum applicants in Germany in 2021 were Syria, Iraq, Afghanistan, and Turkey (12). From a public health perspective, reliable information on specific transmission dynamics and facilitators are essential to provide appropriate and targeted measures of infection prevention to this vulnerable population.

The objective of this study is to gain evidence on past and present SARS-CoV-2 infection rates among refugees by measuring SARS-CoV-2 RNA and specific IgG antibodies upon arrival in Berlin. This study further aims to explore flight-associated risk factors for SARS-CoV-2 infections in order to provide recommendations for improving refugees’ health care environment.

## Materials and methods

### Study design and participants

**Figure 1:**
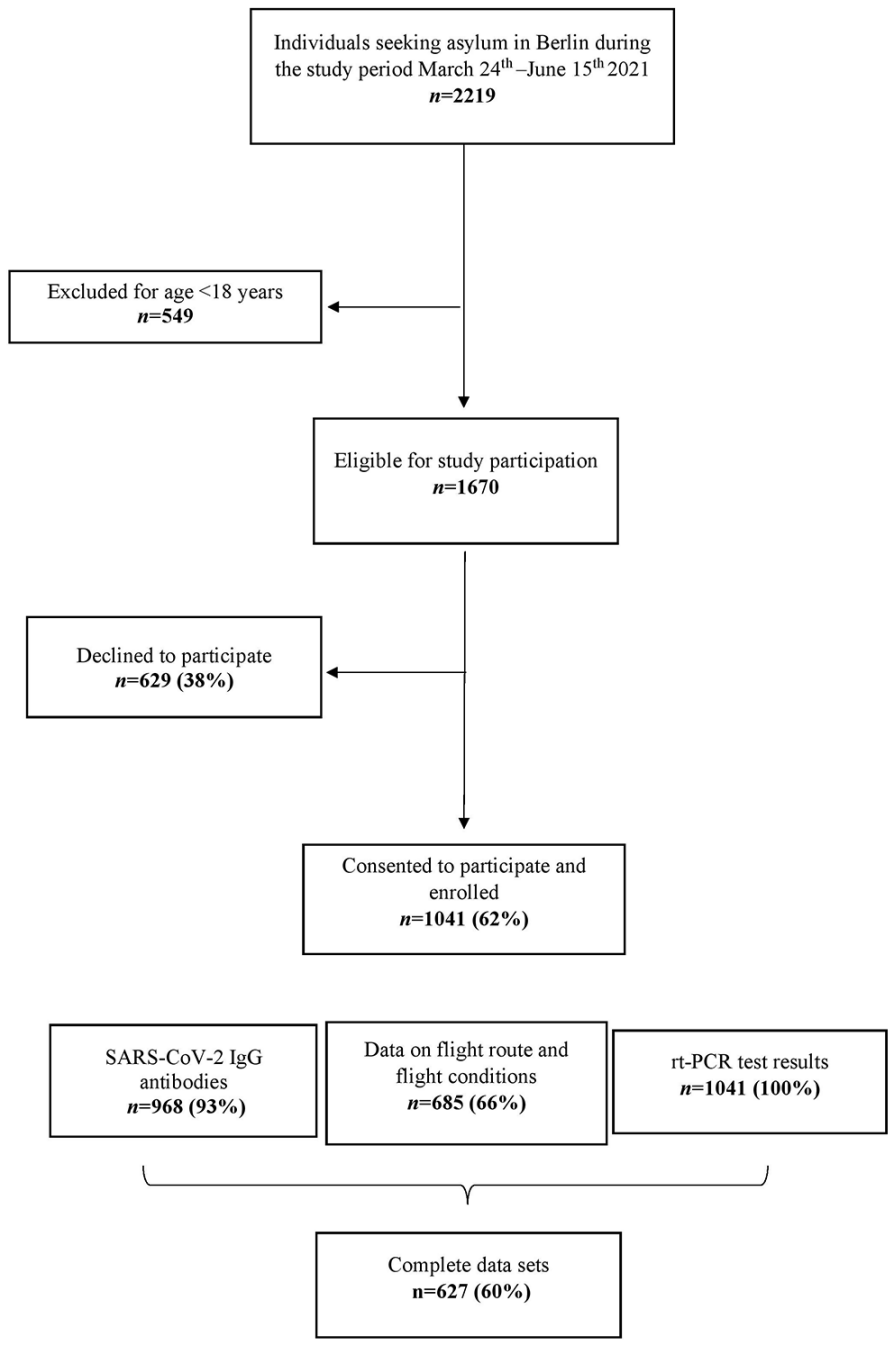
Flowchart of participant enrolment. rt-PCR = reverse transcriptase polymerase chain reaction.

We conducted an observational, cross-sectional study on asylum seekers arriving in Berlin, Germany. Recruitment and data collection took place between March 24th and June 15th 2021. Data collection was integrated into the asylum application process of the federal state Berlin. According to the German Asylum Act (§62 Asylgesetz), every asylum seeker must undergo a medical exam upon arrival. In this manuscript, the term ‘asylum seeker’ defines a legal status of a person who awaits a decision on their residence status in the country of refuge while the term ‘refugee’ commonly refers to a person outside the individual home country due to feared persecution for various reasons (13). In the federal state Berlin, Germany, the Charité – Universitätsmedizin Berlin carries out the health examination for all incoming asylum seekers on behalf of the State Authority for Refugees. The primary goal is to diagnose, treat, and prevent the transmission of acute and chronic diseases, predominantly infectious diseases such as tuberculosis, viral, and parasitic infections. With the spread of the COVID-19 pandemic, a mandatory reverse transcriptase polymerase chain reaction (rt-PCR) test for SARS-CoV-2 was added to the health examination (§28–§31 IfSG) on June 22nd 2020. All individuals (aged ≥18 years) arriving in Berlin and with the aim of applying for asylum were invited to participate in the study (Figure 1).

### Procedures

Data collected at Charité’s medical examination centre for asylum seekers (“Erstaufnahmeuntersuchungsstelle” [EAU]) included medical history, a combined nasal mid-turbinate and throat swab, and the donation of a venous blood sample. Samples were analyzed at the diagnostic laboratory of the Charité. SARS-CoV-2 IgG antibody avidity tests were conducted at Charité Institute of Virology.

Recruitment and data collection were carried out by physicians and in the presence of an interpreter to overcome language barriers or illiteracy hindering study participation. Participants were asked to complete an on-site self-report medical questionnaire (Supplementary material I). A second self-report questionnaire on the participants’ flight route and travel conditions (Supplementary material II) was completed at the State Authority for Refugees (“Landesamt für Flüchtlingsangelegenheiten” [LAF]) in Berlin in the form of a semi-structured interview and with the assistance of trained interpreters. All staff responsible for data collection (both at EAU and LAF) received schooling from research associates prior to data collection. Data was pseudonymized to ensure participants’ privacy. REDCap^®^ electronic data capture tools hosted at the Charité was used to manage data from both questionnaires (14). Study participation had no influence on the process or outcome of the participants’ asylum applications. Ethical approval was granted by the ethics committee of the Charité on March 5th 2021 (EA2/040/21). Data protection and privacy terms were established with the support of the legal department of the Charité and the LAF.

### Measures

Acute SARS-COV-2 infection was assessed using rt-PCR and the LightCycler 480 or cobas 6800/8800 systems from Roche. SARS-CoV-2 specific IgG antibodies were determined using an anti-SARS-CoV-2 S1 IgG assay according to the manufacturer’s instructions (Euroimmun, Lübeck, Germany) and as validated before (15). A titer cut-off value of ≥1.1 was considered when identifying positive serological samples. SARS-CoV-2 IgG antibody avidity was measured using a modified anti-SARS-CoV-2 S1 IgG ELISA (16). An avidity index cut-off value of 60% was set to differentiate between an immune answer to a recent SARS-CoV-2 infection, which occurred during the past 4 weeks, and an infection with a history longer than 4 weeks.

Serological information and data on flight history were combined to categorize individuals into two groups based on their estimated time point of SARS-CoV-2 infection (exposure group I: infection prior to flight; exposure group II: infection during flight). Participants who traveled 8 days or less until registering for asylum in Berlin were assigned to exposure group I. Among this group, infection with SARS-CoV-2 likely occurred prior to flight (*n* = 137). Participants who initiated their flight before January 1st 2020 were assigned to exposure group II. Their infection with SARS-CoV-2 likely occurred during transit as they left their home country before the beginning of the pandemic (*n* = 215). If participants could not be allocated to either group (*n* = 250), antibody avidity testing was conducted among previously infected individuals (*n* = 62). Among those, antibody avidity helped to assign 6 individuals to exposure group I and 30 individuals to exposure group II.

Item construction for both questionnaires was based on surveys regarding health monitoring among asylum seekers and was adapted to the present study context (17, 18). Both questionnaires were devised by an interdisciplinary team including physicians, psychologists, and public health researchers. Prior to data collection, both questionnaires were piloted in March 2021 and revised according to interviewer’s feedback. With questionnaire A, data on past COVID-19 related symptoms, prior SARS-CoV-2 testing, hygiene behaviors, and educational background were collected. The presence of COVID-19 related symptoms (fever, cough, loss of taste and smell, headaches, sore throat, runny nose, fatigue, gastrointestinal complaints, body aches, breathlessness) was assessed regarding the past 24 h, past 14 days, and generally during transit. The frequency of performing hygiene behaviors (social distancing, wearing face masks, and hand washing practices) during flight was assessed using a 4-point Likert-type scale ranging from 0 to 3 (never – always). Using questionnaire B, detailed information concerning flight route, length of stay, duration of stopovers, modes of transport and accommodation, number of people who shared the same vehicle, and members of travel group was assessed chronologically for each transit section. The trial was registered prospectively at ISRCTN registry (Trial number: 17401860).

### Statistical analyses

Analyses were carried out using IBM SPSS Statistics 27. The rate of SARS-CoV-2 infections was calculated as the number of positive tests weighted by the number of tests carried out. Based on data on flight routes, countries of origin were categorized into seven clusters (i.e., geographical regions as flight starting points): Africa, the Balkans, Caucasus, Eastern Europe, Middle East, Western Asia, and Southeast Asia (19). We compared sociodemographic and flight-associated characteristics between participants with and without SARS-CoV-2 IgG antibodies using T-tests and χ2-tests for continuous and categorical variables, respectively. A principal component analysis on hygiene behavior items was performed and a sum score was computed.

Stepwise logistic regression models were used to examine predictors of the presence of SARS-CoV-2 IgG antibodies (i.e., binary outcome). For the subsample of 602 participants with available data on flight conditions, a regression model was calculated including all sociodemographic and selected flight-related study variables (model A). Among participants in exposure group I (*n* = 143), only sociodemographic variables and the hygiene behavior measure were modeled as predictors (model B). Among participants in exposure group II (*n* = 245), flight-related study variables were included as predictors (model C). All logistic regression models were adjusted for age, sex, and educational level. The region of origin with the highest seropositivity rate (i.e., the Balkans) was chosen as the reference category.

## Results

Between March 24th and June 15th 2021, 1670 adult asylum seekers registered at the EAU, of which 1041 (62.3%) agreed to participate in the study (Figure 1). SARS-CoV-2 rt-PCR results were obtained from all participants and for 968 (93.0%) participants within the sample, data on SARS-CoV-2 IgG antibodies was available. Information about flight route and conditions were available from a subset of 685 (65.8%) participants. Two participants had been vaccinated against SARS-CoV-2 before recruitment and were excluded from statistical analysis.

In total, 359 (34.5%) participants were female (view Supplementary material III for a full table of sociodemographic sample characteristics). Mean age was 32.6 years (SD = 10.6) and the average amount of educational years was 8.9 (SD = 5.2). Study participants originated from 43 different countries from Asia, Africa, Europe, and South America. The five most frequent countries of origin were Moldova (*n* = 213, 20.5%), Georgia (*n* = 197, 18.9%), Syria (*n* = 135, 13.0%), Afghanistan (*n* = 118, 11.3%), and Vietnam (*n* = 95, 9.1%). According to their country of origin, participants were clustered into seven regions of origin: Africa (*n* = 68, 6.5%), the Balkans (*n* = 238, 22.9%), Caucasus (*n* = 203, 19.5%), Eastern Europe (*n* = 50, 4.8%), Middle East (*n* = 222, 21.3%), Western Asia (*n* = 26, 9.2%), Southeast Asia (*n* = 153, 14.7%, Supplementary material IV). An acute SARS-CoV-2 infection was diagnosed among 2.8% of the sample (29 of 1041). In this subgroup, 11 (37.9%) participants reported acute symptoms related to COVID-19 in the past 14 days. Four out of 29 PCR-positive tested participant samples were randomized for genotyping. The identified PANGO lineages were B.1.1.7, B.1.1.9, B1.617.2 and one non-VOC variant.

**Figure 2:**
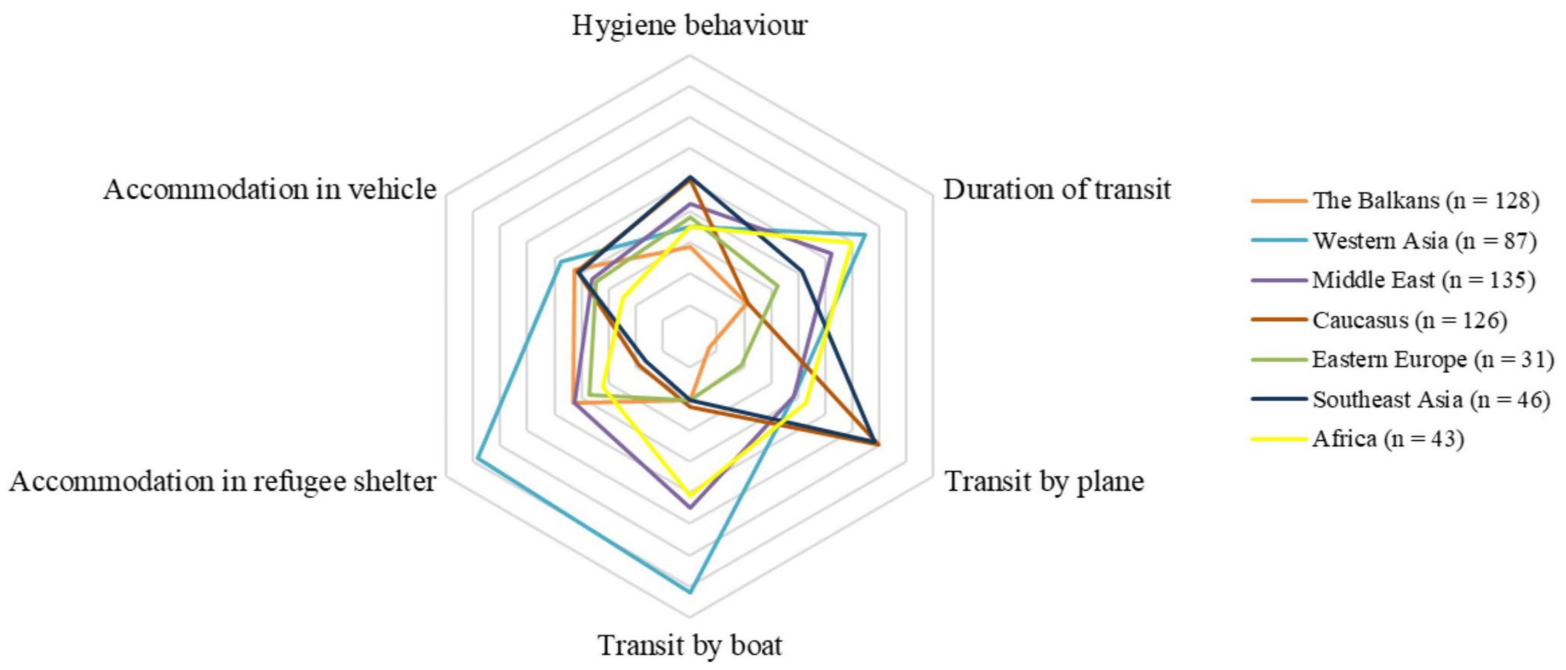
Flight characteristics according to region of origin (*n* = 596).

Averaged flight characteristics for regions of origin are displayed by Figure 2. Mean duration of transit was 546 days (i.e., 1.5 years; SD = 850 days) and sometimes comprised longer stays in places of transit. The average travel group size comprised 1.7 (SD 4.2) people with individuals from Africa, Caucasus, and Southeast Asia being more likely to travel solo. In total, 149 participants (21.7%) reported traveling with children. On average, 30.3% (208 of 686) of participants reported they found accommodation at a refugee shelter during transit. 41.0% (285 of 632) of individuals stayed in a vehicle overnight during at least one transit section. Regarding mode of transport, 17.6% (121 of 686) reported traveling by boat, 45.0% (309 of 686) by plane, and 15.5% (106 of 686) by foot for at least one part of the journey. In total, 296 (28.5%) participants stated they were tested for SARS-CoV-2 at least once in their home country before transit and 450 (43.3%) participants reported being tested at least once during transit.

Of 954 analyzable blood samples, 239 (25.1%) were tested positive for SARS-CoV-2 S1 IgG antibodies. Participant characteristics stratified by seropositivity are presented in Table 1. Participants who tested seropositive were more likely to be female (43.1% [*n* = 103] vs. 32.9% [*n* = 235], *p* = 0.004) and reported a lower educational level (7.8 years [SD = 5.4] vs. 9.3 years [SD = 5.1], *p* < 0.001). Seropositivity rate varied between regions of origin with the Balkans presenting the highest (*n* = 73, 34.4%) and Caucasus presenting the lowest (*n* = 28, 15.8%) seropositivity rate. Among participants who tested seropositive, a higher proportion reported traveling with children (28.5% [*n* = 41] vs. 20.1% [*n* = 98], *p* = 0.03), staying at refugee shelters (42.4% [*n* = 61] vs. 27.7% [*n* = 135], *p* = 0.001), and accomplishing part of their flight by foot (22.2% [*n* = 32] vs. 14.5% [*n* = 71], *p* = 0.03). Furthermore, seropositivity was associated with a lower frequency of traveling by plane (31.3% [*n* = 45] vs. 49.0% [*n* = 239], *p* < 0.001), lower engagement in hygiene behaviors (1.90 [SD = 0.94] vs. 2.13 [SD = 0.83], *p* = 0.001), and lower information seeking on COVID-19 (91.6% [*n* = 219] vs. 95.1% [*n* = 680], *p* = 0.046). Among seropositive tested participants, 27 (11.3%) individuals reported to have experienced COVID-19 related symptoms during their flight.

**Table 1:** Comparison of sociodemographic and flight characteristics of asylum seekers in total and subgroups related to SARS-CoV-2 IgG antibody status.

	Total (*N* = 954)	Seropositive (*n* = 239)	Seronegative (*n* = 715)	Value of *p* (*t*-test)
Mean age, years (SD)	32.8 (10.7)	33.9 (11.4)	32.4 (10.4)	0.08
Sex, female, *n* (%)	338 (35.4)	103 (43.1)	235 (32.9)	<0.01
Mean education, years (SD)	8.9 (5.2)	7.8 (5.4)	9.3 (5.1)	<0.01
Region of origin (*n* = 943)				Value of *p* (*t*-test)
Africa, *n* (%)	69 (7.3)	13 (18.8)	56 (81.2)	
The Balkans, *n* (%)	212 (22.5)	73 (34.4)	139 (65.6)	<0.01^b^
Caucasus, *n* (%)	177 (18.8)	28 (15.8)	149 (84.2)	
Eastern Europe, *n* (%)	48 (5.1)	13 (27.1)	35 (72.9)	
Middle East, *n* (%)	213 (22.6)	44 (20.7)	169 (79.3)	
Southeast Asia, *n* (%)	85 (9.0)	17 (20.0)	68 (80.0)	
Western Asia, *n* (%)	139 (14.7)	47 (33.8)	92 (66.2)	
Duration of transit				Value of *p* (*t*-test or chi-square test)
Mean duration of transit (*n* = 459), days (SD)	564 (872)	508 (712)	580 (914)	0.41
Stay in Berlin before registration (*n* = 943), *n* (%)	253 (26.5)	59 (24.7)	194 (27.2)	0.44
Travel group				
Mean number of people in travel group (*n* = 211), ᴓ (SD)	1.7 (4.2)	1.8 (2.8)	1.6 (4.4)	0.80
Mean number of people to share sleeping place (*n* = 636), ᴓ (SD)	4.4 (6.6)	4.4 (5.1)	4.4 (7.0)	0.98
Traveling with children (*n* = 632), *n* (%)	139 (22.0)	41 (28.5)	98 (20.1)	0.03
Mode of transport for at least one part of the flight (*n* = 632)^a^				
Boat, *n* (%)	116 (18.4)	25 (17.4)	91 (18.6)	0.73
Plane, *n* (%)	284 (44.9)	45 (31.3)	239 (49.0)	<0.01
By foot, *n* (%)	103 (16.3)	32 (22.2)	71 (14.5)	0.03
Accommodation during transit (*n* = 632)^a^				
In refugee shelter, *n* (%)	196 (31.0)	61 (42.4)	135 (27.7)	<0.01
In vehicle, *n* (%)	262 (40.8)	61 (41.5)	201 (40.6)	0.85
Prior testing for SARS-CoV-2 (*n* = 954)				
≥1 tests for SARS-CoV-2 during transit, *n* (%)	414 (43.4)	97 (40.6)	317 (44.3)	0.31
≥1 test for SARS-CoV-2 in home country, *n* (%)	267 (28.0)	64 (26.8)	203 (28.4)	0.63
Hygiene behavior (*n* = 954)				
Hygiene behavior sum score (SD)	2.07 (0.86)	1.90 (0.94)	2.13 (0.83)	<0.01
Information seeking on COVID-19, *n* (%)	899 (94.2)	219 (91.6)	680 (95.1)	0.046

^a^Multiple answers were possible as participants were requested to report the mode of transport used for each travel section. ^b^*t*-Test for The Balkans vs. Caucasus: *t*(384) = −4.36, *p* = <0.01.

**Figure 3:**
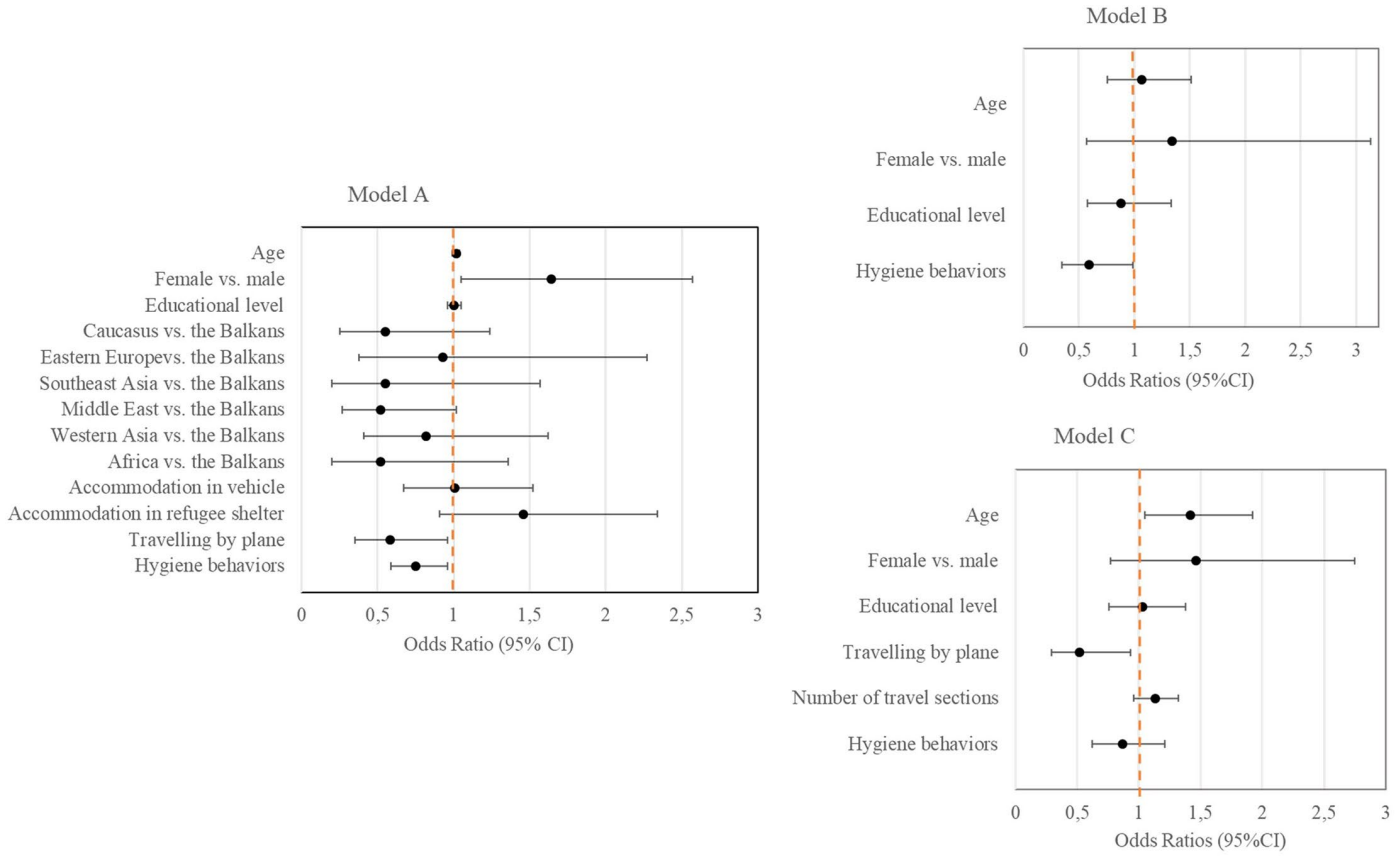
Factors associated with the presence of SARS-CoV-2 IgG antibodies (Forest plots). In model A, factors related to seropositivity are presented for the entire subgroup of participants with available information on flight characteristics (*n* = 602). In model B, factors related to seropositivity are presented for participants in exposure group I whose potential infection happened before flight (*n* = 143). In model C, factors related to seropositivity are presented for participants in exposure group II whose potential infection happened during transit (*n* = 245).

In logistic regression analysis on flight-associated factors related to seropositivity in the sample of 602 participants (Figure 3, model A), women were 1.6-times more likely to show SARS-CoV-2 IgG antibodies compared to men (OR [95%CI] = 1.64 [1.05–2.57]; *p* = 0.03). Traveling by plane (vs. never using a plane during transit; OR [95%CI] = 0.58 [0.35–0.96]; *p* = 0.04) and a higher frequency of hygiene behaviors (OR [95%CI] = 0.75 [0.59–0.96]; *p* = 0.02) were significantly associated with a lower probability of seropositivity. None of the regions of origin showed significant associations with SARS-CoV-2 specific antibodies when compared to the reference category the Balkans (Supplementary material V–VII, Model A–C).

Seropositivity rate among the subgroup whose potential infection likely happened before flight (exposure group I) was 25.2% and 31.8% among the subgroup with a potential infection during transit (exposure group II). Logistic regression for exposure group I (Figure 3, model B) revealed that engaging in hygiene behaviors was related to a lower likelihood for seropositivity (OR [95%CI] = 0.59 [0.35–0.99]; *p* = 0.045). Among participants in exposure group II (Figure 3, model C), older age was associated with a higher probability for seropositivity (OR [95%CI] = 1.42 [1.05–1.92]; *p* = 0.02). Regarding flight characteristics, traveling by plane was associated with a lower probability for seropositivity (OR [95%CI] = 0.52 [0.29–0.93]; *p* = 0.03). Both, the number of travel sections as an indicator of duration of transit and the hygiene behavior score were not significantly associated with seropositivity rate in this subgroup.

## Discussion

Our findings highlight the increased exposure to SARS-CoV-2 among asylum seekers arriving in Berlin in 2021. Around a quarter of the study population presented SARS-CoV-2 specific IgG antibodies. Seroprevalence in the German general population was lower at the same time and varied between 4.1% to 13.1% in four German communities (20). Most studies conducted so far described increased infection estimates among refugee and asylum seeking populations compared to community-based samples based on acute infections detected by rt-PCR (5). In the present study, the observed incidence rate of acute SARS-CoV-2 infection of 2.8% was higher than the range of 0.1% to 2.1% reported by a review summarizing results on incidence risk of SARS-CoV-2 infection among refugees in non-outbreak settings (5). To further put this incidence rate into perspective, official records for the German population reported a 7-day incidence rate of 0.12% on the 24th of March (119.3 acute infections per 100,000 people on the first day of data collection) and 0.014% on the 15th of June (13.8 acute infections per 100,000 people on the last day of data collection) for the state of Berlin (21). While these rates are lower than those reported for incoming asylum seekers, direct comparisons between these groups are difficult due to the very different testing strategies, alternative reporting schemes, fundamental differences in health quality and health literacy, and the lack of a matched sample population. Comparative data on hospitalization and lethality rates among refugees and asylum seekers paint a mixed picture, leaving the burden of COVID-19 on refugees unclear (22). Beyond cross-sectional data, serological surveys on past infections are a valuable tool to gain insight into the extent of exposure to SARS-CoV-2, especially for younger populations with expected higher proportions of asymptomatic infection, such as asylum seekers and refugees.

In the present study, frequent engagement in hygiene behaviors was associated with a 25.0% lower chance of presenting SARS-CoV-2 specific antibodies. Among participants with an estimated infection prior to flight, a risk reduction of 41% for prior SARS-CoV-2-infection was connected to the consistent adherence to hygiene behaviors. In contrast, serostatus was not related to hygiene behaviors among participants who likely were infected during flight (exposure group II). When exposed to uncontrollable environmental factors during flight, such as overcrowding, the impact of preventive hygiene behavior on the individual infection risk might be limited. When living under more controllable conditions (e.g., in one’s home country), engagement in hygiene behaviors might be more feasible and potentially develop a stronger protective effect. Health literacy is associated with health-related behavior, but can be compromised among refugees (23). Therefore, public health measures might not address refugees’ health needs in the same manner as they do in the resident population. As health literacy is a strong modifiable risk factor for socioeconomic health disparities, it should be a target of public health initiatives attempting to minimize health inequity (24).

Seropositivity rate differed substantially across regions of origin and was associated with staying in a refugee shelter during transit. Yet, in multivariate analysis, accommodation in a refugee shelter did not show statistical significance. Congested living settings were suggested to be a key factor for unrestrained virus transmission by observational (25, 26) and mathematical modeling studies (6). The epidemic potential of SARS-CoV-2 among refugees arises particularly through outbreak situations in overcrowded living arrangements. In those settings, effective containment strategies including self-isolation, surveillance testing and vaccinations are often not feasible. Outbreaks of COVID-19 have been described not only in German refugee shelters (25) but also in other densely populated housing facilities, such as migrant worker residences and shelters for the homeless (26, 27). Hence, refugees’ vulnerability to COVID-19 can be interpreted partly as a result of their increased exposure to overcrowded accommodation, identifying a focus for action on infection control.

Female sex and low educational level were linked to higher seropositivity rates in our data. These findings were unexpected and raise several points of discussion. The increased risk of testing seropositive among women might be attributed to their closer contact to children as principal caretakers and children’s function as drivers of asymptomatic transmission (28). Accordingly, participants who reported traveling with children were more likely to test seropositive. Women tend to flee in larger groups due to safety issues, cultural norms, and family ties and receive priority for shelter and shared housing compared to male refugees. These increased points of contact might heighten the probability of transmission for female refugees compared to (young) men who travel alone more frequently (29, 30). Another possible explanation for the observed effect may arise from the lack of equal opportunities for women. In many countries and cultures, it is common for women to remain at home to carry out household and family duties instead of pursuing education (31). This in turn may have a negative impact on their health literacy and increase their risk for infection. Due to the exploratory nature of the presented data, causal conclusion on gender-specific determinants of hygiene behaviors cannot be drawn but should be further investigated.

The lowered chances for seropositivity observed for individuals traveling by plane might be due to comparably controllable travel conditions as well as due to strict pandemic-related boarding regulations to prevent SARS-CoV-2 infections.

The study population’s diverse composition of places of origin reflects that living situations worldwide are marked by political conflict and economic insecurity, forcing people to leave their home country to seek international protection (32). Due to the comparably large sample size, our data provide a meaningful descriptive portrait of refugees’ travel conditions and complements the sparse information on this hard-to-reach population. Of note, study participants from Vietnam (*n* = 95) seemed to constitute a unique subgroup with the highest proportion of female participants (58.9%), of whom, by the time of study participation, 45 individuals (79.0%) were pregnant at advanced stages. Although approximately 70.0% reported to have traveled by plane, mean duration of transit was about 1.5 years. Together with the high percentage of pregnant women in this subgroup, the discrepancy might reflect a particular aspect in German legislation to apply for asylum at a later stage of pregnancy.

It is important to take into account the limitations of our study. First, it is not possible to determine precisely when and where exactly participants were infected with SARS-CoV-2 due to the cross-sectional design. Although combining antibody avidity with descriptive data presents an advanced approach to differentiate between recent and former infections, it can only provide an approximation of the infection interval. Additionally, as antibody titers drop over time, some participants might have been infected with SARS-CoV-2 previously but were tested seronegative in ELISA. Secondly, the study participation rate among asylum seekers was 62.0%. Individuals with an exhausting travel history or experiences of trauma might have been less willing to participate in the study. In addition, 356 (34.0%) of study participants did not appear at the second study site (LAF) where the questionnaire on flight route was administered. We assumed that the dropout rate rather reflected a delay or cancelation of the asylum application procedure than a study participation refusal. Still, selection bias could affect the generalizability of presented findings. Third, the seropositivity rate might be underestimated due to the exclusion of minors in our study. In Germany, more than half of registered asylum applications between January and October 2021 were submitted by minors. Lastly, social desirability bias as well as memory recall bias could have influenced responding, especially when reporting the adherence to hygiene behaviors and recalling longer transit histories, respectively. Some participants might have withheld information on their transit because they feared negative consequences on later residency status decisions.

In conclusion, our findings underline the increased exposure of asylum seekers to SARS-CoV-2 infections and identify correlates of an infection before or during their flight. Individual factors, such as hygiene behaviors, as well as environmental factors, such as accommodation in refugee shelters, were found to contribute to refugees’ increased vulnerability toward a SARS-CoV-2 infection. When integrating these findings, the improvement of living and hygiene conditions during flight should be target candidates of future public health interventions. For establishing health equality even during a pandemic, future research should illuminate other sources of disadvantage for individuals on the move such as legal restrictions regarding health care, language barriers and stigma.

## Data availability statement

The datasets presented in this article are not readily available because participants did not consent to their data being distributed to external parties. Requests to access the datasets should be directed to the corresponding author.

## Ethics statement

The studies involving human participants were reviewed and approved by Ethics Committee of the Charité, Universitätsmedizin Berlin [approval granted on 05.03.2021 (EA2/040/21)]. The patients/participants provided their written informed consent to participate in this study.

## Author contributions

AB and LB contributed equally to the conception of the study, study design, data collection, data management, analysis, and manuscript writing. JK contributed to the statistical analysis and interpretation of data. VC contributed to the management, sequencing, and analysis of probe material. NB and JS contributed equally to the conception, design, and supervision of the study. All authors contributed to the article and approved the submitted version.

## Funding

The study was conducted as part of the “Bundesweites Forschungsnetz Angewandte Surveillance und Testung” [B-FAST (national network for research “applied surveillance and testing”)]. Funding for B-FAST was provided by the German Ministry for Education and Research (BMBF). The BMBF was not involved in planning the study’s design, data collection, analysis, or manuscript writing.

## Conflict of interest

VC was employed by Labor Berlin – Charité Vivantes GmbH.

The remaining authors declare that the research was conducted in the absence of any commercial or financial relationships that could be construed as a potential conflict of interest.

## Publisher’s note

All claims expressed in this article are solely those of the authors and do not necessarily represent those of their affiliated organizations, or those of the publisher, the editors and the reviewers. Any product that may be evaluated in this article, or claim that may be made by its manufacturer, is not guaranteed or endorsed by the publisher.

## References

[1] The UN Refugee Agency. Global trends in forced displacement (2021). Available at: https://www.unhcr.org/globaltrends.html (Accessed November 22, 2021).

[2] Vonen HD Olsen ML Eriksen SS Jervelund SS Eikemo TA. Refugee camps and COVID-19: can we prevent a humanitarian crisis? Scand J Public Health. (2021) 49:27–8. doi: 10.1177/1403494820934952, PMID: 32522120

[3] World Health Organization. Report on the health of refugees and migrants in the WHO European region: No public health without refugee and migrant health. Denmark: World Health Organization (2018).

[4] Kluge HH Jakab Z Bartovic J D'Anna V Severoni S. Refugee and migrant health in the COVID-19 response. Lancet. (2020) 395:1237–9. doi: 10.1016/S0140-6736(20)30791-1, PMID: 32243777 PMC7270965

[5] Hintermeier M Gencer H Kajikhina K Rohleder S Hövener C Tallarek M. SARS-CoV-2 among migrants and forcibly displaced populations: a rapid systematic review. J Migr Health. (2021) 4:100056. doi: 10.1016/j.jmh.2021.100056, PMID: 34151312 PMC8205550

[6] Truelove S Abrahim O Altare C Lauer SA Grantz KH Azman AS. The potential impact of COVID-19 in refugee camps in Bangladesh and beyond: a modeling study. PLoS Med. (2020) 17:e1003144. doi: 10.1371/journal.pmed.1003144, PMID: 32544156 PMC7297408

[7] Fouad FM McCall SJ Ayoub H Abu-Raddad LJ Mumtaz GR. Vulnerability of Syrian refugees in Lebanon to COVID-19: quantitative insights. Confl Heal. (2021) 15:13. doi: 10.1186/s13031-021-00349-6, PMID: 33673855 PMC7934989

[8] Islam MM Yunus MY. Rohingya refugees at high risk of COVID-19 in Bangladesh. Lancet Glob Health. (2020) 8:e993–4. doi: 10.1016/S2214-109X(20)30282-5, PMID: 32593313 PMC7316456

[9] Agency TUNR. UNHCR figures at a glance (2020). Available at: https://www.unhcr.org/uk/figures-at-a-glance.html (Accessed October 31, 2021).

[10] Bundesamt für Migration und Flüchtlinge. Aktuelle Zahlen 1. Halbjahr 2021 (2021). Available at: https://www.bamf.de/DE/Themen/Statistik/Asylzahlen/asylzahlen-node.html (Accessed November 22, 2021).

[11] Guadagno L. Migrants and the COVID-19 pandemic: an initial analysis. International Organization for Migration (IOM) Geneva (2020)

[12] Flüchtlingsangelegenheiten Lf. Aktuelle Ankunftszahlen (2021). Available at: https://www.berlin.de/laf/ankommen/aktuelle-ankunftszahlen/artikel.625503.php (Accessed November 22, 2021).

[13] Douglas P Cetron M Spiegel P. Definitions matter: migrants, immigrants, asylum seekers and refugees. J Travel Med. (2019) 26:26. doi: 10.1093/jtm/taz005, PMID: 30753575

[14] Harris PA Taylor R Minor BL Elliott V Fernandez M O'Neal L. The REDCap consortium: building an international community of software platform partners. J Biomed Inform. (2019) 95:103208. doi: 10.1016/j.jbi.2019.103208, PMID: 31078660 PMC7254481

[15] Aziz NA Corman VM Echterhoff AK Müller MA Richter A Schmandke A. Seroprevalence and correlates of SARS-CoV-2 neutralizing antibodies from a population-based study in Bonn, Germany. Nat Commun. (2021) 12:2117. doi: 10.1038/s41467-021-22351-5, PMID: 33837204 PMC8035181

[16] Schwarz T Tober-Lau P Hillus D Helbig ET Lippert LJ Thibeault C. Delayed antibody and T-cell response to BNT162b2 vaccination in the elderly, Germany. Emerg Infect Dis. (2021) 27:2174–8. doi: 10.3201/eid2708.211145, PMID: 34102097 PMC8314803

[17] Biddle L Menold N Bentner M Nöst S Jahn R Ziegler S. Health monitoring among asylum seekers and refugees: a state-wide, cross-sectional, population-based study in Germany. Emerg Themes Epidemiol. (2019) 16:3. doi: 10.1186/s12982-019-0085-2, PMID: 31316579 PMC6613239

[18] Skogberg N Koponen P Tiittala P Mustonen K-L Lilja E Snellman O. Asylum seekers health and wellbeing (TERTTU) survey: study protocol for a prospective total population health examination survey on the health and service needs of newly arrived asylum seekers in Finland. BMJ Open. (2019) 9:e027917. doi: 10.1136/bmjopen-2018-027917, PMID: 30962242 PMC6500271

[19] Frontex. Migratory map – routes (2021). Available at: https://frontex.europa.eu/we-know/migratory-map/ (Accessed December 13, 2021).

[20] Gornyk D Harries M Glöckner S Strengert M Kerrinnes T Heise J-K. SARS-CoV-2 seroprevalence in Germany. Dtsch Arztebl Int. (2021) 118:824–31. doi: 10.3238/arztebl.m2021.0364, PMID: 35191825 PMC8888869

[21] Landesamt für Gesundheit und Soziales Berlin. COVID-19 in Berlin, Fallzahlen und Indikatoren – Gesamtübersicht (2021). Available at: https://www.berlin.de/lageso/gesundheit/infektionskrankheiten/corona/tabelle-indikatoren-gesamtuebersicht/ (Accessed March 21, 2023).

[22] Hayward SE Deal A Cheng C Crawshaw A Orcutt M Vandrevala TF. Clinical outcomes and risk factors for COVID-19 among migrant populations in high-income countries: a systematic review. J Migr Health. (2021) 3:100041. doi: 10.1016/j.jmh.2021.100041, PMID: 33903857 PMC8061095

[23] Wångdahl J Lytsy P Mårtensson L Westerling R. Health literacy among refugees in Sweden - a cross-sectional study. BMC Public Health. (2014) 14:1030. doi: 10.1186/1471-2458-14-1030, PMID: 25278109 PMC4195944

[24] Stormacq C van den Broucke S Wosinski J. Does health literacy mediate the relationship between socioeconomic status and health disparities? Integrative review. *Health Promot In*t. (2019) 34:e1–e17. doi: 10.1093/heapro/day062, PMID: 30107564

[25] Jahn R Hintermeier M Bozorgmehr K. SARS-CoV-2 attack rate in reception and accommodation centres for asylum seekers during the first wave: systematic review of outbreak media reports in Germany. J Migr Health. (2022) 5:100084. doi: 10.1016/j.jmh.2022.100084, PMID: 35136866 PMC8815301

[26] Roederer T Mollo B Vincent C Nikolay B Llosa AE Nesbitt R. Seroprevalence and risk factors of exposure to COVID-19 in homeless people in Paris, France: a cross-sectional study. Lancet Public Health. (2021) 6:e202–9. doi: 10.1016/S2468-2667(21)00001-3, PMID: 33556328 PMC7993986

[27] Chew MH Koh FH Wu JT Ngaserin S Ng A Ong BC. Clinical assessment of COVID-19 outbreak among migrant workers residing in a large dormitory in Singapore. J Hosp Infect. (2020) 106:202–3. doi: 10.1016/j.jhin.2020.05.034, PMID: 32492454 PMC7261446

[28] Mehta NS Mytton OT Mullins EW Fowler TA Falconer CL Murphy OB. SARS-CoV-2 (COVID-19): what do we know about children? A systematic review. Clin Infect Dis. (2020) 71:2469–79. doi: 10.1093/cid/ciaa556, PMID: 32392337 PMC7239259

[29] Freedman J. Sexual and gender-based violence against refugee women: a hidden aspect of the refugee “crisis”. Reprod Health Matters. (2016) 24:18–26. doi: 10.1016/j.rhm.2016.05.003, PMID: 27578335

[30] Arsenijević J Burtscher D Ponthieu A Severy N Contenta A Moissaing S. "I feel like I am less than other people": health-related vulnerabilities of male migrants travelling alone on their journey to Europe. Soc Sci Med. (2018) 209:86–94. doi: 10.1016/j.socscimed.2018.05.038, PMID: 29807316

[31] Salikutluk Z Menke K. Gendered integration? How recently arrived male and female refugees fare on the German labour market. JFamRes. (2021) 33:284–321. doi: 10.20377/jfr-474

[32] Cummings C Pacitto J Lauro D Foresti M. Why people move: understanding the drivers and trends of migration to Europe. London: Overseas Development Institute (2015).

