# Supplementary material for: Seropositivity and flight-associated risk factors for SARS-CoV-2 infection among asylum seekers arriving in Berlin, Germany – a cross-sectional study
Source: Front Public Health. 2023 Jun 12;11:1134546. doi: 10.3389/fpubh.2023.1134546 (PMC10291620; doi:10.3389/fpubh.2023.1134546)
Supplement: Supplementary file 1 [file Data_Sheet_1.pdf]

**Supplementary appendix for**

# Seropositivity and Flight-Associated Risk Factors for SARS-CoV-2 Infection among Asylum Seekers arriving in Berlin, Germany
# – A Cross-Sectional Study-

## Table of Content


I. Questionnaire A. Primary medical data ........ 2

II. Questionnaire B. Primary data on flight route and conditions ........ 4

III. Supplementary Table 1. Sociodemographic and flight characteristics by region of origin (n=1 041). ........ 6

IV. Supplementary Table 2. Distribution of home countries clustered into regions of origin. ........ 9

V. Supplementary Table A. Logistic regression model on independent variable ‘serostatus’, n=602. ........ 10

VI. Supplementary Table B. Logistic regression model on independent variable ‘serostatus’, n=143. ........ 10

VII. Supplementary Table C: Logistic regression model on independent variable ‘serostatus’, n=245. ........ 10

## I. Questionnaire A. Primary medical data

Participants were required to complete a short survey at the EAU medical centre. Items were focused on COVID-19 related symptoms experienced across the past two weeks and during refugees' flight from their home country, SARS-CoV-2 testing history, access to information regarding the pandemic, and the individual's adherence to basic infection prevention measures over the past two weeks. Due to the existing language barrier, items were translated and presented verbally by the present physicians and translators. Answers were recorded digitally.

Date and time of survey completion: __________________

Study ID: __________________

Which of the following statements is true regarding the study participant's status upon arrival at the EAU centre?

- Consultation in the EAU centre directly after arrival in Berlin (antigen rapid test was negative)
- Consultation in the EAU centre after isolation (prior positive antigen rapid test + positive PCR test)
- Consultation in the EAU centre after quarantine (i.e. due to contact with a positive individual, but own PCR test was negative)
- Other: __________________________________

### Demographics

1. Date of arrival in Berlin: _______________
2. How many years of education did you undergo? _______________
3. Which educational degree have you attained?
   - None
   - Elementary school
   - Middle school
   - High school
   - Vocational training
   - University degree
   - Other
   - I don't know / no answer

### Symptoms and Diagnostics

4. Did you have one or more of the following symptoms in the past 48 hours? (Multiple answers possible)
   - I did not have any symptoms
   - Fever
   - Runny nose
   - Fatigue
   - Sore limbs or muscle pain
   - Gastrointestinal issues
   - Headache
   - Change in sense of smell or taste
   - Cough
   - Sore throat
   - Shortness of breath
5. Did you have one or more of the following symptoms in the past 14 days? (Multiple answers possible)

- I did not have any symptoms
- Fever
- Runny nose
- Fatigue
- Sore limbs or muscle pain
- Gastrointestinal issues
- Headache
- Change in sense of smell or taste
- Cough
- Sore throat
- Shortness of breath

6. Did you have one or more of the following symptoms for at least 24 hours during your transit to Germany? (Multiple answers possible)
    - I did not have any symptoms
    - Fever
    - Runny nose
    - Fatigue
    - Sore limbs or muscle pain
    - Gastrointestinal issues
    - Headache
    - Change in sense of smell or taste
    - Cough
    - Sore throat
    - Shortness of breath

7. Have you informed yourself about COVID-19? If so, which sources of information did you use?
    - Yes, I informed myself …
    - No, I had no interest in informing myself
    - No, I had no possibility to inform myself
    - I don't know

    7.1. If you informed yourself, which sources did you use? (Multiple answers possible)

    - Radio
    - Television
    - Newspaper/magazines
    - Podcasts
    - YouTube (or similar streaming platforms)
    - Internet search
    - Social media (i.e. Facebook, Twitter)
    - Informational material (i.e. flyers, posters, official notices)
    - Medical experts (i.e. during a medical consultation)
    - Through family / friends

8. Did you get tested for COVID-19 <u>during</u> your transit?
    - Yes
    - No

    8.1 If yes, date of the test: ________________

    8.2 What was the result of the test?
    - Positive
    - Negative
    - I don't know

    8.3 Was a further test carried out during your transit?

    → *Questions 8.1 and 8.2 repeated for test two, three and four if applicable*

9. Did you get tested for COVID-19 <u>prior</u> to your transit (while still in your home country)?
    - Yes
    - No

9.1 If yes, date of the test: _______________
9.2 What was the result of the test?
- Positive
- Negative
- I don’t know

9.3 Was a further test carried out prior to your transit?
→ *Questions 9.1 and 9.2 repeated for test two, three and four if applicable*

## II. Questionnaire B. Primary data on flight route and conditions

During the processing of an asylum application, detailed information about every refugee’s background is documented by administrators of the LAF. Questionnaire B was completed during the asylum registration process. Administrators queried the participants about their transit route to Berlin, including details about mode of transport, duration of transit, type of shelter, and number of fellow travellers. Participants were asked to work backwards through their journey, from their most recent stopover to their initial departure. Information provided by the study participants was guaranteed to have no impact on the further procedures involved in their asylum application and was solely used for the purpose of the study.

Date of survey completion: __________________

Study ID: __________________

Was the survey completed?

a. Yes
b. Not completed
c. Refused

When did you leave your place of origin and begin your journey to Germany? ____________________

Did you transit through one or more countries?

d. Yes
e. No

Step 1:

1.1 Country: __________________
1.2 Town/City: _________________
1.3 Type of Stay
- Stopover
- Part of travel route
- Part of travel route completed with support
- Country of origin

1.4 Duration of Stay: ___________________ days/weeks/months
1.5 Accommodation type
- Refugee camp / tent
- Refugee camp / shelter
- Private accommodation / house / flat

- Payed accommodation
- On the streets / in the open
- Vehicle
- Prison
- Quarantine facility
- Other

1.6 Number of individuals, who shared the accommodation: ___________

1.7 Mode of transport used to arrive:

- Bus
- Lorry
- Car
- Train
- Boat
- Airplane
- By foot
- Other/I don't know

1.8 Number of individuals who shared the mode of transportation: ______________

1.9 Number of adults, with whom you were travelling: _______________

1.10 Number of children, with whom you were travelling: _______________

Step 2: All questions from step 1 were repeated for each stage / transit during flight. A maximum of nine stages were reported.

## III. Supplementary Table 1. Sociodemographic and flight characteristics by region of origin (n=1 041).

| | **Africa** n=68 (6.5) | **The Balkans** n=238 (22.9) | **Caucasus** n=203 (19.5) | **Eastern Europe** n=50 (4.8) | **Middle East** n=222 (21.3) | **Southeast Asia** n=96 (9.2) | **Western Asia** n= 153 (14.7) | **Other** n = 11 (1.1) | **Total** n = 1 041 |
|---|---|---|---|---|---|---|---|---|---|
| Age, ⌀ (SD), years | 29.8 (7.3) | 36.0 (12.4) | 34.2 (8.9) | 35.5 (12.9) | 30.0 (9.1) | 28.3 (7.6) | 32.0 (11.3) | 31.3 (16.4) | 32.6 (10.6) |
| Sex, female, n (%) [a] | 10 (14.7) | 103 (43.5) | 53 (26.1) | 23 (46.0) | 62 (27.9) | 57 (59.4) | 48 (31.4) | 3 (27.3) | 359 (24.5) |
| Years of education, ⌀ (SD) | 10.8 (5.2 | 4.3 (4.5) | 12.0 (2.9) | 10.8 (4.4) | 9.5 (4.7) | 11.2 (2.4) | 7.5 (5.7) | 12.3 (3.3) | 8.9 (5.2) |
| Seropositivity rate, n (%) (n=954) | 14 (20.6) | 73 (34.4) | 28 (15.8) | 13 (27.1) | 44 (20.7) | 17 (20.0) | 47 (32.4) | 4 (36.4) | 239 (25.1) |
| PCR positive tested cases, n (%) | 0 (0.0) | 6 (2.5) | 4 (2.0) | 1 (2.0) | 4 (81.8) | 2 (2.1) | 12 (7.8) | 0 (0.0) | 29 (2.8) |
| Duration of transit | | | | | | | | | |
| Duration of transit, ⌀ (SD), days (n=609) | 951 (1,056 | 163 (273) | 152 (461) | 442 (669) | 814 (1,159) | 533 (671) | 1085 (786) | 1186 (964) | 546 (850) |
| Stay in Berlin before registration, n (%) (n=1027) | 11 (16.2) | 21 (8.9) | 16 (7.9) | 7 (14.0) | 103 (46.6) | 63 (65.6) | 47 (30.7) | 3 (27.3) | 271 (26.1) |
| Travel group | | | | | | | | | |
| No. of people in travel group, ⌀ (SD) (n=211) | 0.5 (1.0) | 4.1 (8.0) | 0.4 (0.6) | 1.7 (2.2) | 1.5 (2.9) | 0.3 (0.4) | 2.0 (2.5) | - | 1.7 (4.2) |

| | **Africa**<br><br>**n=68 (6.5)** | **The Balkans**<br><br>**n=238 (22.9)** | **Caucasus**<br><br>**n=203 (19.5)** | **Eastern Europe**<br><br>**n=50 (4.8)** | **Middle East**<br><br>**n=222 (21.3)** | **Southeast Asia**<br><br>**n=96 (9.2)** | **Western Asia**<br><br>**n= 153 (14.7)** | **Other**<br><br>**n = 11 (1.1)** | **Total**<br><br>**n = 1 041** |
|---|---|---|---|---|---|---|---|---|---|
| No. of people to share sleeping place with, ø (SD) (n=691) | 4.4 (7.8) | 4.3 (4.2) | 4.1 (5.7) | 3.1 (2.1) | 3.8 (3.4) | 4.3 (6.3) | 6.0 (12.7) | 2.8 (1.1) | 4.3 (6.6) |
| Travelling with children, n (%) (n=686) | 5 (10.4) | 51 (25.2) | 21 (14.9) | 8 (23.5) | 33 (22.3) | 0 (0.0) | 28 (28.9) | 3 (60.0) | 149 (21.7) |
| Transport media on transit | | | | | | | | | |
| Boat, n (%) (n=686) | 14 (29.2) | 0 (0.0) | 3 (2.1) | 0 (0.0) | 46 (31.1) | 0 (0.0) | 57 (58.8) | 1 (20.0) | 121 (17.6) |
| Plane, n (%) (n=686) | 19 (29.6) | 7 (4.8) | 117 (83.0) | 6 (17.6) | 63 (42.6) | 47 (69.1) | 47 (48.5) | 3 (60.0) | 309 (45.0) |
| Walking, n (%) (n=686) | 5 (10.4) | 1 (0.7) | 2 (1.4) | 1 (2.9) | 61 (41.2) | 0 (0.0) | 34 (25.1) | 2 (40.0) | 106 (15.5) |
| Accommodation during transit | | | | | | | | | |
| In refugee shelter, n (%) (n=686) | 9 (18.8) | 51 (35.2) | 10 (7.1) | 10 (29.4) | 50 (33.8) | 2 (2.9) | 73 (75.3) | 3 (60.0) | 208 (30.3) |
| In vehicle, n (%) (n=632) | 10 (20.4) | 63 (43.8) | 65 (45.8) | 13 (38.2) | 54 (36.0) | 27 (37.0) | 50 (51.5) | 3 (50.0) | 285 (41.0) |
| Prior testing for SARS-CoV-2 | | | | | | | | | |
| ≥1 test for SARS-CoV-2 during flight, n (%) | 28 (41.2) | 71 (30.0) | 118 (58.1) | 19 (38.0) | 93 (41.9) | 12 (12.5) | 101 (66.0) | 8 (72.7) | 450 (43.3) |
| ≥1 test for SARS-CoV-2 in home country, n (%) | 16 (23.5) | 53 (22.4) | 120 (59.1) | 16 (32.0) | 43 (19.4) | 5 (5.2) | 41 (26.8) | 2 (18.2) | 296 (28.5) |
| Health behaviour | | | | | | | | | |

| | **Africa** n=68 (6.5) | **The Balkans** n=238 (22.9) | **Caucasus** n=203 (19.5) | **Eastern Europe** n=50 (4.8) | **Middle East** n=222 (21.3) | **Southeast Asia** n=96 (9.2) | **Western Asia** n= 153 (14.7) | **Other** n = 11 (1.1) | **Total** n = 1 041 |
|---|---|---|---|---|---|---|---|---|---|
| Hygiene behaviour score, $\bar{x}$ (SD) | 1.99 (0.89) | 1.85 (0.96) | 2.31 (0.71) | 2.05 (0.95) | 2.14 (0.78) | 2.32 (0.77) | 1.98 (0.88) | 2.73 (0.47) | 2.10 (0.86) |
| Informed themselves about SARS-CoV-2, n (%) | 63 (92.6) | 214 (90.0) | 200 (98.5) | 49 (98.0) | 215 (96.8) | 93 (96.9) | 136 (88.9) | 11 (100.0) | 981 (94.2) |

## IV. Supplementary Table 2. Distribution of home countries clustered into regions of origin.

| Region of Origin | Total | Home Countries |
|---|---|---|
| Africa | 63 | Algeria (n=10), Benin (n=2), Burkina Faso (n=1), Cameroon (n=4), Ethiopia (n=4), Guinea (n=5), Kuwait (n=2), Liberia (n=2), Libya (n=2), Morocco (n=13), Mozambique (n=1), Nigeria (n=7), Sierra Leone (n=2), Somalia (n=2), Sudan (n=3), Tunisia (n=2) |
| The Balkans | 238 | Albania (n=1), Bosnia (n=5), Kosovo (n=1), Moldova (n=213), Serbia (n = 18) |
| Caucasus | 203 | Armenia (n=3), Azerbaijan (n=3), Georgia (n=197) |
| Eastern Europe | 50 | Belarus (n=5), Russia (n=25), Ukraine (n=20) |
| Middle East | 228 | Egypt (n=8), Iraq (n=19), Jordan (n=1), Lebanon (n=9), Palestine (n=10), Syria (n=135), Turkey (n=46) |
| Southeast Asia | 96 | Vietnam (n=95), Philippines (n=1) |
| West Asia | 153 | Afghanistan (n=118), Iran (n=15), Pakistan (n=13), Yemen (n=6), Usbekistan (n=1) |
| Other | 10 | Not known (n=7), Venezuela (n=3) |

## V. Supplementary Table A. Logistic regression model on independent variable 'serostatus', n=602.

| | Odds Ratio | 95% CI | *p* |
|---|---|---|---|
| Age | 1.02 | 0.99-1.04 | .08 |
| Female vs. male | 1.64 | 1.05-2.57 | .03 |
| Educational level | 1.00 | 0.96-1.05 | .92 |
| Region of origin | | | |
| Caucasus vs. The Balkans | 0.55 | 0.25-1.24 | .15 |
| Eastern Europe vs. The Balkans | 0.93 | 0.38-2.27 | .87 |
| Southeast Asia vs. The Balkans | 0.55 | 0.20-1.57 | .27 |
| Middle East vs. The Balkans | 0.52 | 0.27-1.02 | .06 |
| Western Asia vs. The Balkans | 0.82 | 0.41-1.62 | .56 |
| Africa vs. The Balkans | 0.52 | 0.20-1.36 | .18 |
| Flight-associated risk factors | | | |
| Accommodation in vehicle | 1.01 | 0.67-1.52 | .97 |
| Accommodation in refugee shelter | 1.46 | 0.91-2.34 | .11 |
| Travelling by plane | 0.58 | 0.35-0.96 | .04 |
| Hygiene behaviour score | 0.8 | 0.59-0.96 | .02 |

## VI. Supplementary Table B. Logistic regression model on independent variable 'serostatus', n=143.

| | Odds Ratio | 95% CI | *p* |
|---|---|---|---|
| Age | 1.07 | 0.75-1.51 | .71 |
| Female vs. male | 1.34 | 0.57-3.13 | .51 |
| Educational level | 0.88 | 0.58-1.34 | .55 |
| Hygiene behaviour score | 0.59 | 0.35-0.99 | .05[a] |

[a] p=.045

## VII. Supplementary Table C: Logistic regression model on independent variable 'serostatus', n=245.

| | Odds Ratio | 95% CI | *p* |
|---|---|---|---|
| Age | 1.42 | 1.05-1.92 | .02 |
| Female vs. male | 1.46 | 0.77-2.75 | .24 |
| Educational level | 1.03 | 0.76-1.38 | .87 |
| Travelling by plane | 0.52 | 0.29-0.93 | .03 |
| Number of travel sections | 1.13 | 0.96-1.32 | .15 |
| Hygiene behaviour score | 0.87 | 0.62-1.21 | .41 |
